# *Lactobacillus gasseri* LA39 Activates the Oxidative Phosphorylation Pathway in Porcine Intestinal Epithelial Cells

**DOI:** 10.3389/fmicb.2018.03025

**Published:** 2018-12-11

**Authors:** Jun Hu, Libao Ma, Wenyong Zheng, Yangfan Nie, Xianghua Yan

**Affiliations:** ^1^State Key Laboratory of Agricultural Microbiology, College of Animal Sciences and Technology, Huazhong Agricultural University, Hubei, China; ^2^The Cooperative Innovation Center for Sustainable Pig Production, Hubei, China; ^3^Hubei Provincial Engineering Laboratory for Pig Precision Feeding and Feed Safety Technology, Hubei, China

**Keywords:** *Lactobacillus gasseri* LA39, intestinal epithelium, oxidative phosphorylation, iTRAQ, piglets

## Abstract

Intestinal microbial interactions with the host epithelium have important roles in host health. Our previous data have suggested that *Lactobacillus gasseri* LA39 is the predominant intestinal *Lactobacillus* in weaned piglets. However, the regulatory role of *L. gasseri* LA39 in the intestinal epithelial protein expression in piglets remains unclear. In the present study, we conducted comparative proteomics approach to investigate the intestinal epithelial protein profile alteration caused by *L. gasseri* LA39 in piglets. The expressions of 15 proteins significantly increased, whereas the expressions of 13 proteins significantly decreased in the IPEC-J2 cells upon *L. gasseri* LA39 treatment. Bioinformatics analyses, including COG function annotation, GO annotation, and KEGG pathway analysis for the differentially expressed proteins revealed that the oxidative phosphorylation (OXPHOS) pathway in IPEC-J2 cells was significantly activated by *L. gasseri* LA39 treatment. Further data indicated that two differentially expressed proteins UQCRC2 and TCIRG1, associated with the OXPHOS pathway, and cellular ATP levels in IPEC-J2 cells were significantly up-regulated by *L. gasseri* LA39 treatment. Importantly, the *in vivo* data indicated that oral gavage of *L. gasseri* LA39 significantly increased the expression of UQCRC2 and TCIRG1 and the cellular ATP levels in the intestinal epithelial cells of weaned piglets. Our results, both *in vitro* and *in vivo*, reveal that *L. gasseri* LA39 activates the OXPHOS pathway and increases the energy production in porcine intestinal epithelial cells. These findings suggest that *L. gasseri* LA39 may be a potential probiotics candidate for intestinal energy production promotion and confers health-promoting functions in mammals.

## Introduction

Growing evidence has suggested that intestinal microbes have critical roles in intestinal homeostasis and host health (Sommer and Backhed, 2013). Intestinal microbes have been found to function in host immune defense system maturation (Ivanov et al., 2009), intestinal epithelium differentiation (Sommer and Backhed, 2013), and nutrients digestion (Backhed et al., 2007). However, intestinal microbial dysbiosis can cause host gastrointestinal diseases (Borody and Khoruts, 2011), such as irritable bowel syndrome, inflammatory bowel disease, and diarrhea. An increasing number of studies have investigated the health-promoting roles of probiotics (Ventura et al., 2009; Lemon et al., 2012). According to the Food and Agriculture Organization of the United Nations and the WHO (FAO/WHO), probiotics are defined as “live microorganisms which when administered in adequate amounts confer a health benefit on the host” (Hill et al., 2014). The intestinal microbe-host interaction has become a research focus in microbiology (Kim et al., 2010). Thus, exploring the potential regulatory role of probiotic candidates in intestinal epithelium is of great significance.

Proteomics has been used in intestinal microbiota-host interaction research (Olivares et al., 2012; Siciliano and Mazzeo, 2012; van de Guchte et al., 2012; Luo et al., 2013; Ayllón et al., 2017). Olivares et al., using 2-dimensional gel electrophoresis (2-DE) and MALDI-TOF-TOF peptide fingerprinting, showed that oral gavage of rats with *Bifidobacterium longum* modulated the jejunal proteome in rats (Olivares et al., 2012). Luo et al. demonstrated that the probiotic, *Enterococcus faecium* altered the proteome in the intestinal mucosa of the broilers. Using 2-D fluorescence difference gel electrophoresis (DIGE), they exhibited that the chickens fed with *E. faecium* used less nutrient and energy while responding to the immune and antioxidant stresses (Luo et al., 2013). Ayllón et al., 2017 suggested that both host and pathogen factors were responsible for the commensal or infectious character of *Campylobacter jejuni* in different hosts, using sequential window acquisition of all theoretical fragment ion-mass spectrometry (SWATH-MS). Currently, a proteomics approach, termed isobaric tags for relative and absolute quantification (iTRAQ), has been widely performed to evaluate the alteration of protein expression profiles because of its several advantages, including high sensitivity, high-throughput, and accuracy (Treumann and Thiede, 2010). It has been used by us to investigate the alteration of protein expression profiles in weaned piglets (Fan et al., 2017; Hu et al., 2017). However, the iTRAQ strategy has not been used to evaluate the role of intestinal microbes in intestinal epithelial protein expression regulation.

Given the similarities in the anatomy and nutritional physiology between pigs (*Sus scrofa*) and human beings, the pig has been used as a research model (Vodicka et al., 2005; Maxmen, 2012; Meurens et al., 2012; Nielsen et al., 2014). Considering that members of the *Lactobacillus* genus may be potential probiotic candidates (Kleerebezem et al., 2010; van Baarlen et al., 2013), we mainly focused on the health-promoting roles of a *Lactobacillus* species. Our previous data showed that *Lactobacillus gasseri* LA39 was a predominant intestinal *Lactobacillus* in the weaned piglets (Hu et al., 2016). *L. gasseri* LA39 was found to produce gassericin A bacteriocin (Kawai et al., 1994) which is active against several pathogenic bacteria (Kawai et al., 2001), suggesting a probiotic potential of *L. gasseri* LA39. However, the regulatory role of *L. gasseri* LA39 in the expression of the intestinal epithelial proteins remains unclear. The present study was designed to investigate the potential role of *L. gasseri* LA39 in intestinal epithelium regulation using iTRAQ strategy. Our findings revealed that *L. gasseri* LA39 could activate the oxidative phosphorylation (OXPHOS) pathway in porcine intestinal epithelial cells showed. The ATP levels were also significantly increased with *L. gasseri* LA39 treatment. Our results suggest that *L. gasseri* LA39 may facilitate increasing intestinal energy production and confer health-promoting functions in mammals. Our data also further suggest the important interactions of gut microbes with host physiology in mammals.

## Materials and Methods

### *In vitro* Assay of Bacterial Adhesion to Intestinal Porcine Epithelial Cell Line From the Jejunum (IPEC-J2) Cells

IPEC-J2 cells were cultured in DMEM/F12 (Gibco, 11320-033) containing 10% FBS (Gibco, 10099-141) in 5% CO_2_ at 37°C. The *L. gasseri* LA39 (JCM 11657) was obtained from the Japan Collection of Microorganisms (JCM). It was cultured in de Man, Rogosa and Sharpe (MRS) medium at 37°C in an anaerobic incubator. The viable IPEC-J2 cell count was performed by trypan blue staining and subsequent microscopic counting, and the viable *L. gasseri* LA39 count was performed with methylene blue staining, followed by microscopic counting. To investigate the bacterial adhesion to IPEC-J2 cells, we used a method combining bacterial fluorescein-5-isothiocyanate (FITC) labeling and intestinal epithelial cellular plasma membrane labeling by wheat germ agglutinin (WGA). Briefly, viable *L. gasseri* LA39 were co-incubated with FITC dye (Thermo Fisher Scientific, F1906) for 1 h at room temperature, and then washed 3 times with PBS. Subsequently, FITC-labeled *L. gasseri* LA39 were added into the IPEC-J2 cell medium for 2 h or 4 h, the initial ratios of *L. gasseri* LA39 numbers to IPEC-J2 cells numbers were 1:1, 10:1, or 100:1. Following co-culture, IPEC-J2 cells were washed three times with PBS to remove the *L. gasseri* LA39 that has not adhered and then fixed with 4% paraformaldehyde solution. After washing three times with PBS buffer, the IPEC-J2 cells were incubated with 5 μg/ml WGA (Thermo Fisher Scientific, W32466) at room temperature for 10 min. Finally, FITC fluorescence (excitation 488 nm) and WGA fluorescence (excitation 633 nm) were detected by a laser scanning confocal microscope (ZEISS, LSM 880). The IPEC-J2 cells stained with WGA were shown by red color and the *L. gasseri* LA39 (adhered) stained with FITC dye were shown by green color in the fluorescence images. The numbers of adhered bacterial cells were calculated using the ratios of *L. gasseri* LA39 numbers to IPEC-J2 cells numbers in the fluorescence images. The final relative numbers of adhered bacterial cells (fold change) were normalized to the numbers of adhered bacterial cells (FITC-labeled *L. gasseri* LA39 were added into IPEC-J2 cell medium for 2 h, and the initial ratio of *L. gasseri* LA39 numbers to IPEC-J2 cells numbers was 1:1).

### Co-culture Assay of IPEC-J2 Cells and *L. gasseri* LA39

The *L. gasseri* LA39 used in the present study is a facultative anaerobe and can also survive under aerobic conditions, thereby such an *in vitro* model is appropriate to investigate the intestinal epithelial responses to *L. gasseri* LA39. Considering that the numbers of adhered bacterial cells were the largest when the co-culture assay was conducted for 4 h and the initial ratio of bacterial cells numbers to IPEC-J2 cells numbers was 100:1 in our data, we chose this co-culture protocol (including the co-culture time and initial ratios for interaction) to investigate the intestinal epithelial cellular responses to *L. gasseri* LA39. The IPEC-J2 cells and the viable *L. gasseri LA39* count was performed by staining as mentioned previously. Subsequently, viable *L. gasseri* LA39 were added into the IPEC-J2 cell medium for 4 h, the initial ratio of *L. gasseri* LA39 numbers to IPEC-J2 cells numbers was 100:1. Similar dilutions of DMEM/F12 medium were used as vehicle controls.

### Protein Extraction, Trypsin Digestion, and iTRAQ Labeling

Upon completion of the co-culture assay, the IPEC-J2 cells were washed three times with sterile PBS buffer to remove the *L. gasseri* LA39. The IPEC-J2 cells were frozen with liquid nitrogen immediately and then lysed in the “lysis buffer (8 M Urea, 40 mM Tris–HCl with 1 mM PMSF, 2 mM EDTA, and 10 mM DTT, pH 8.5)” (Li et al., 2018). “After centrifugation at 25,000 *g* at 4°C for 20 min, the supernatant obtained was reduced with 10 mM DTT at 56°C for 1 h, and then alkylated with 55 mM iodoacetamide (IAM) in the dark at room temperature for 45 min” (Huang et al., 2018). Following further centrifugation with 25,000 *g* at 4°C, the supernatant was quantified by Bradford method. The protein solution (100 μg) was digested using Trypsin Gold. Following trypsin digestion, “the peptides were desalted, vacuum-dried, and dissolved in 30 μl of 0.5 M TEAB and labeled by the iTRAQ Reagent 8-plex Kit. The peptides labeled with different reagents were combined, and then desalted and vacuum-dried” (Li et al., 2018).

### Peptide Fractionation, High Performance Liquid Chromatography (HPLC), and Mass Spectrometer Detection

The peptides fractionation, high performance liquid chromatography (HPLC), and mass spectrometer detection were performed using the previously described procedures (Wang et al., 2018).

### Bioinformatics Analyses

The raw MS/MS data was converted into the MGF format using the Proteome Discoverer. The peptide spectrum from raw data will be pre-processed to obtain the peptide spectrum of high quality by filtering out these peptide spectrums (ionic charge > 7). “Proteins were identified by the Mascot search engine” (Weatherly et al., 2005) run against the Uniprot database for *Sus scrofa*. “At least one unique peptide was essential for the identified protein.” The parameters are shown in the Table 1. “An automated software, named IQuant, was conducted to analyze the labeled peptides with isobaric tags quantitatively” (Wen et al., 2014). The main IQuant quantitation parameters are shown in Table 2. False discovery rate (FDR) was used to control the confidence of peptides and proteins as previously described (Savitski et al., 2015; Hu Z. et al., 2018).

**Table 1:** Mascot search parameters.

Item	Value
Type of search	MS/MS ion search
Enzyme	Trypsin
Fragment mass tolerance	0.05 Da
Mass values	Monoisotopic
Variable modification	Oxidation (M), iTRAQ8plex (Y)
Peptide mass Tolerance	20 ppm
Fixed modification	Carbamidomethyl (C), iTRAQ8plex (N-term), iTRAQ8plex (K)
Database	Uniprot database for *Sus scrofa*

**Table 2:** IQuant quantitation parameters.

Item	Value
Quant peptide	Use All unique peptide
Quant number	At least one unique spetra
Normalization	VSN
Protein Ratio	Weighted average
Statistical Analysis	Permutation Tests

Based on some previous iTRAQ analyses (Rhein et al., 2009; Ren et al., 2013; Huang et al., 2015; Shi et al., 2015; Hu et al., 2017), “a ratio of 1.2-fold ( > 1.20 or < 0.833) with a *q*-value < 0.05 were chosen as the cutoff for up-regulated or down-regulated expression, respectively.” To better detect the differentially expressed proteins, “we further defined those proteins significantly altered in at least two replicates as differentially expressed proteins as previously described” (Zhu et al., 2009; Long et al., 2016). “Functional annotations of identified proteins were conducted by COG and GO, respectively.” “We performed a hyper-geometric test to obtain target GO terms in the GO enrichment analysis using our previously described procedures” (Hu et al., 2017). “KEGG enrichment analysis was also performed to identify the KEGG pathways preferentially affected by treatment using our previously described procedures” (Hu et al., 2017).

### *In vivo* Assay for Piglets and Sample Collection

*Lactobacillus gasseri* LA39 was cultured in MRS medium at 37°C. 100 crossbred piglets (Landrace × Yorkshire) with similar birth weight were randomly divided into two groups. Piglets in the same group were randomly divided into 5 pens, and 10 piglets per pen. The piglets in control (Ctrl) group were oral administrated with a vehicle (sterile PBS, 2 ml) once a day from the age of 6–20 days. The piglets in *L. gasseri* LA39 (LG) group were oral administrated with a bacterial suspension (2 ml, 10^8^ CFU/ml *L. gasseri* LA39 in PBS) once a day from the age of 6–20 days. All the piglets were early-weaned at 21 days of age. Each piglet was randomly chosen from each pen at 26 days of age and these piglets selected from Ctrl group (5 piglets) and LG group (5 piglets) were slaughtered. The intestinal tissues were collected from the approximately middle positions in intestinal tracts, including duodenum, jejunum, and ileum, respectively, to reduce sample variability as previously described (Hu et al., 2017). Piglets handling protocols were approved by the Institutional Animal Care and Use Committee of Huazhong Agricultural University. The methods were carried out in accordance with the approved guidelines.

### Western Blotting

The whole cell lysates (WCLs) of IPEC-J2 cells and the WCLs of intestinal epithelial cells from weaned piglets used for western blotting were prepared using RIPA lysis buffer (Sangon Biotech, C500005). Western blotting was conducted by our previously described methods (Hu et al., 2017). Below are the antibodies used in the western blotting: horseradish peroxidase (HRP)-conjugated secondary goat anti-rabbit antibodies (Santa Cruz Biotechnology, sc-2004), UQCRC2 antibody (Abcam, ab203832), TCIRG1 antibody (Proteintech, 12649-1-AP), and GAPDH (ABclone Technology, AC001).

### Measurement of Relative mRNA Expression of Proteins

Total RNA was extracted from the IPEC-J2 cells and intestinal epithelial cells of weaned piglets. Subsequently, cDNA was generated by the PrimeScript TM RT reagent Kit (Takara, RR047A). The relative mRNA expression of these proteins (UQCRC2, TCIRG1, and GAPDH) was measured using qRT-PCR. The specific primers used in qRT-PCR were shown in Table 3. qRT-PCR was conducted using our previously described procedures (Hu J. et al., 2018).

**Table 3:** Specific primers for UQCRC2, TCIRG1, and GAPDH genes.

Gene name		Primers sequences
UQCRC2	Forward	5′-AGCCATCCACGGTCCTTCA-3′
	Reverse	5′-GCTTGCTGCCATTGACTTCC-3′
TCIRG1	Forward	5′-ACGCTTTGTGGTGGATGTTC-3′
	Reverse	5′-CAGGCGATCCGACTCTTCTT-3′
GAPDH	Forward	5′-CCTTCATTGACCTCCACTACAT-3′
	Reverse	5′-GGATCTCGCTCCTGGAAGA-3′

### Measurement of Cellular ATP Levels

The total cellular ATP contents in IPEC-J2 cells and intestinal epithelial cells (including duodenum, jejunum, and ileum) from weaned piglets were measured by an ATP determination kit (Thermo Scientific, A22066) according to the manufacturer’s protocol. The protein concentration of the WCLs was measured by the protein assay kit (Thermo Scientific, 23227). The ATP concentration (nmol/L) was normalized to the total protein concentration (mg/L) of WCLs.

### Statistical Analyses

**FIGURE 1:**
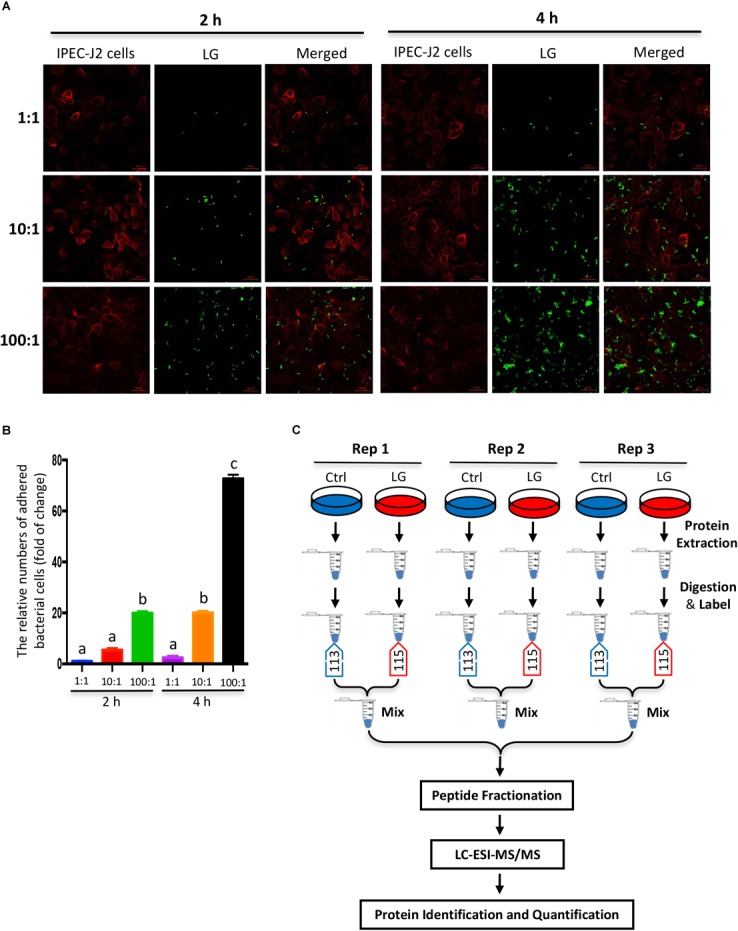
Experimental design and schematic workflow for iTRAQ assay. **(A)** *In vitro* assay of bacterial adhesion to IPEC-J2 cells was shown by fluorescence staining. FITC-labeled *L. gasseri* LA39 (green) were added into IPEC-J2 cell medium for 2 or 4 h and the initial ratios of *L. gasseri* LA39 numbers to IPEC-J2 cell numbers were 1:1, 10:1, or 100:1. The plasma membranes of IPEC-J2 cells were labeled by wheat germ agglutinin (WGA) dye (red). **(B)** The relative numbers of adhered bacterial cells to IPEC-J2 cells. Data are represented as mean ± SEM (*n* = 3). Different letters above the bars denotes a significant difference among the groups. **(C)** Schematic workflow for the iTRAQ assay as described in Materials and Methods.

Statistical analyses were conducted by GraphPad Prism (version 6.0c) software. Two-way analysis of variance (ANOVA) and adjustments for multiple comparisons was conducted for Figure 1B. The Student’s *t*-test was conducted to compare the differences between the two groups in Figures 5C–F, 6. The data are shown as mean ± SEM (ns, not significant, ^∗^*p* < 0.05, ^∗∗^*p* < 0.01).

## Results

### Identification of the Differentially Expressed Proteins in IPEC-J2 Cells by Co-culturing With *L. gasseri* LA39

To investigate the effects of *L. gasseri* LA39 on the porcine intestinal epithelial cells, we used a co-culture method by which live *L. gasseri* LA39 were added into the IPEC-J2 cells medium. The results of the adherence assay indicated that the numbers of adhered bacterial cells were the largest when the co-culture was conducted for 4 h and the initial ratio of bacterial cell numbers to IPEC-J2 cell numbers was 100:1 (Figures 1A,B). Thus, we used the iTRAQ strategy, where live *L. gasseri* LA39 were added into the IPEC-J2 cell medium for 4 h at 100:1 ratio to compare the intestinal epithelial cell protein profiles between the *L. gasseri* LA39 (LG) group and the control (Ctrl) group in the co-culture assay. The workflow for the iTRAQ is shown in Figure 1C. In total, three biological replicates of IPEC-J2 cells from LG and Ctrl groups were used in this study. The tryptic peptides were labeled with isobaric iTRAQ tags (LG: 115; Ctrl: 113), and these labeled peptides were detected using liquid chromatography-electrospray ionization-tandem mass spectrometry (LC-ESI-MS/MS) method.

**FIGURE 2:**
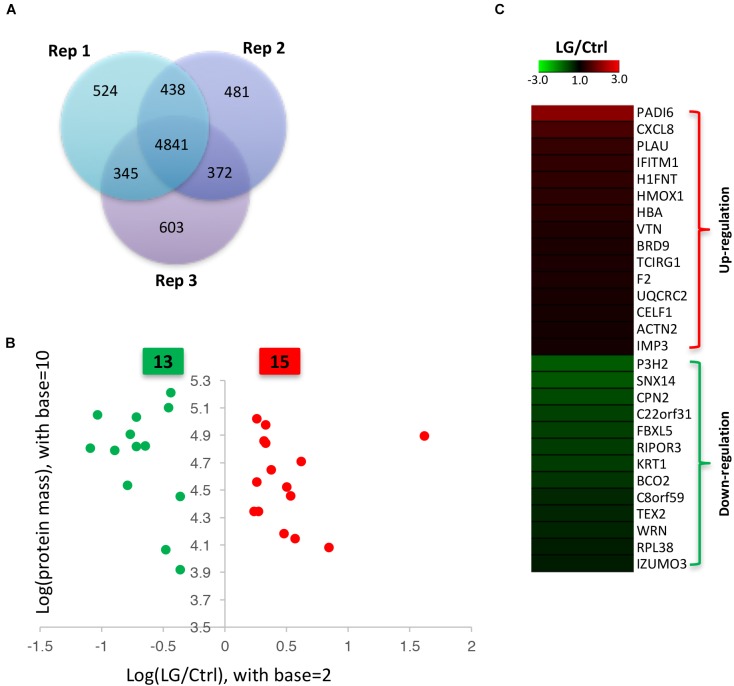
Identification of differentially expressed proteins in IPEC-J2 cells upon *L. gasseri* LA39 treatment using iTRAQ strategy. **(A)** Venn diagrams for the quantified proteins in the three biological replicates. **(B)** Scatter plot analysis based on the differentially expressed proteins. The red spots and green spots indicate the significantly up-regulated proteins and significantly down-regulated proteins, respectively. **(C)** Heat map for the differentially expressed proteins. The values of the colors in the heat map indicate the mean iTRAQ ratio (fold change; LG/Ctrl) in protein expression.

Our data from iTRAQ indicated that a total of 6148, 6132, and 6161 proteins were quantified in the three biological replicates, respectively (Figure 2A). Of these quantified proteins, 4841 proteins were commonly identified in all the three biological replicates (Figure 2A). Based on those previous iTRAQ studies (Rhein et al., 2009; Ren et al., 2013; Huang et al., 2015; Shi et al., 2015; Hu et al., 2017), an iTRAQ ratio of 1.2-fold ( > 1.20 or < 0.833) with a *q*-value < 0.05 was chosen as the cutoff for up-regulated or down-regulated expression, respectively. To better detect the differentially expressed proteins, we further defined these proteins significantly altered in at least two replicates as the differentially expressed proteins as previously described (Zhu et al., 2009; Long et al., 2016). As shown in the scatter plot and heat map, the expressions of 15 proteins significantly increased, whereas the expressions of 13 proteins significantly decreased in IPEC-J2 cells after *L. gasseri* LA39 treatment (Figures 2B,C). The detailed information for differentially expressed proteins is listed in Table 4.

**Table 4:** Differentially expressed proteins quantified by iTRAQ strategy in IPEC-J2 cells.

Accession	Gene name	Protein name	Ratio (Rep 1)	Ratio (Rep 2)	Ratio (Rep 3)	Ratio (mean)
**Up-regulated proteins (LG/ Ctrl)**					
K9J6L7	TCIRG1	V-type proton ATPase subunit a	1.268^*^	1.274^*^	1.225^*^	1.26
P04185	PLAU	Urokinase-type plasminogen activator	1.553^*^		1.519^*^	1.54
P32394	HMOX1	Heme oxygenase 1	1.380^*^	1.418^*^	1.457^*^	1.42
F1SUQ9	PAD I6	Peptidyl arginine deiminase 6		2.889^*^	3.262^*^	3.08
F1RPD4	UQCRC2	Cytochrome b-c1 complex subunit 2	1.141	1.250^*^	1.242^*^	1.21
P01965	HBA	Hemoglobin subunit alpha	1.315^*^	1.320^*^	1.554^*^	1.4
F1RGC4	IFITM1	Interferon-induced transmembrane protein 1	1.720^*^	1.450^*^	1.286	1.49
Q19AZ8	F2	Prothrombin	1.111^*^	1.417^*^	1.235^*^	1.25
F1SID9	CELF1	CUGBP Elav-like family member 1	1.202^*^	1.303^*^	1.100^*^	1.2
I3L9F8	VTN	Vitronectin	1.159^*^	1.405^*^	1.323^*^	1.3
F1SGU6	H1FNT	H1 histone family member N, testis specific	1.232	1.558^*^	1.556^*^	1.45
A0A0B8RW18	BRD9	Bromodomain containing 9	1.058	1.404^*^	1.330^*^	1.26
P26894	CXCL8	Interleukin-8	1.777^*^	1.680^*^	1.950^*^	1.8
F1SJ73	IMP3	U3 small nucleolar ribonucleoprotein	1.267^*^	1.058	1.203^*^	1.18
F1RHL9	ACTN2	Alpha-actinin-2	1.235^*^	1.333^*^	1.018	1.2
**Down-regulated proteins (LG/ Ctrl)**					
F1SGG3	KRT1	Keratin 1	0.588^*^	0.426^*^	0.818^*^	0.61
F1S5E2	FBXL5	F-box and leucine rich repeat protein 5		0.519^*^	0.665^*^	0.59
F1SM94	BC02	Beta-carotene oxygenase 2		0.677^*^	0.603^*^	0.64
I3LR87	C22orf31	Chromosome 22 open reading frame 31	0.573^*^	0.668^*^	0.507^*^	0.58
F1RSL6	TEX2	Testis expressed 2	0.683^*^	0.707^*^	0.800	0.73
F1RX70	WRN	Werner syndrome RecQ like helicase	0.795^*^	0.757^*^	0.656	0.74
I3LNH2	RIPOR3	RIPOR family member 3		0.583^*^	0.635^*^	0.61
F1SP77	IZUM03	IZUMO family member 3	0.825	0.727^*^	0.802^*^	0.78
F2Z568	RPL38	Ribosomal protein L38	0.824^*^	0.763^*^	0.749^*^	0.78
I3LF89	CPN2	Carboxypeptidase N subunit 2	0.442^*^	0.528^*^	0.645^*^	0.54
F1SFH5	P3H2	Prolyl 3-hydroxylase 2	0.505^*^		0.440^*^	0.47
F1RXB9	C8orf59	Chromosome 8 open reading frame 59	0.706^*^	0.728^*^	0.725	0.72
F1S0H9	SNX14	Sorting nexin 14	0.463^*^	0.576	0.419^*^	0.49

*^∗^q-value < 0.05.*

### COG Annotation and Cellular Compartment Analyses of the Differentially Expressed Proteins

**FIGURE 3:**
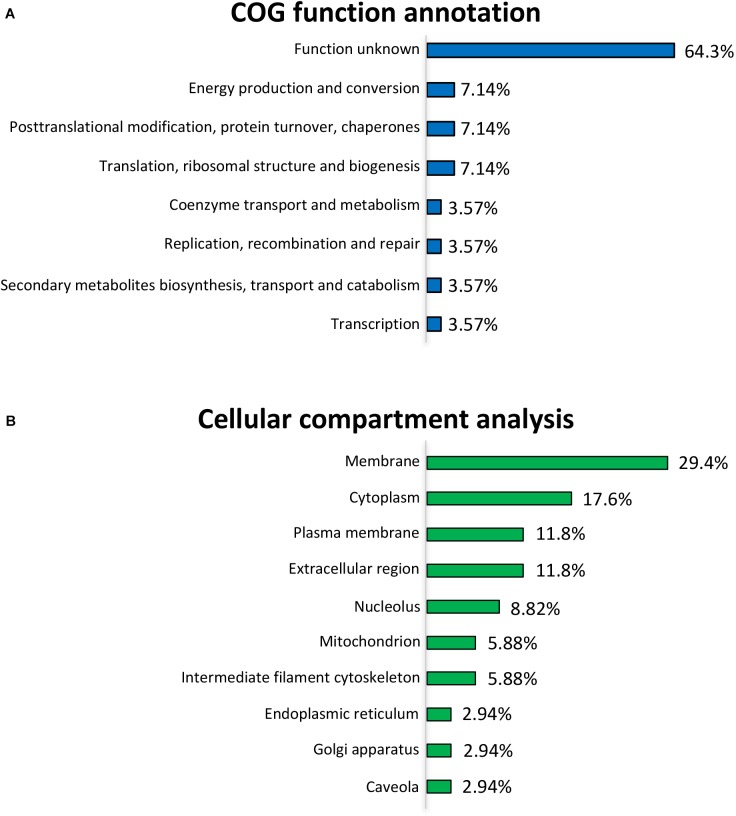
COG function annotation and cellular compartment analyses for the differentially expressed proteins. **(A)** COG function annotation for the differentially expressed proteins. The relative proportions of the COG terms are shown on the right of the corresponding columns. **(B)** Cellular compartment analysis for the differentially expressed proteins. The relative proportions of the GO terms for cellular compartment are shown on the right of the corresponding columns.

To further uncover the effects of *L. gasseri* LA39 on the protein profiles of the IPEC-J2 cells, we used the COG annotation and GO annotation to analyze the differentially expressed proteins. The results showed that these COG functions, including energy production and conversion; post-translational modification, protein turnover, chaperones; translation, ribosomal structure and biogenesis were mainly annotated in the differentially expressed proteins (Figure 3A). Cellular compartment analysis from GO annotation indicated that the differentially expressed proteins were mainly located in the membrane, cytoplasm, plasma membrane, extracellular region, nucleolus, mitochondrion, intermediate filament cytoskeleton, endoplasmic reticulum, Golgi apparatus, and caveolae (Figure 3B). The subcellular localization in plasma membrane and extracellular region suggested a positive intestinal epithelial response to the extracellular *L. gasseri* LA39. The COG function “energy production and conversion” and the cellular compartment “mitochondrion” annotated in the differentially expressed proteins, suggested that *L. gasseri* LA39 treatment may have a regulatory role in the mitochondrial energy metabolism of porcine intestinal epithelial cells.

### Biological Process and Molecular Function Analyses of Differentially Expressed Proteins

**FIGURE 4:**
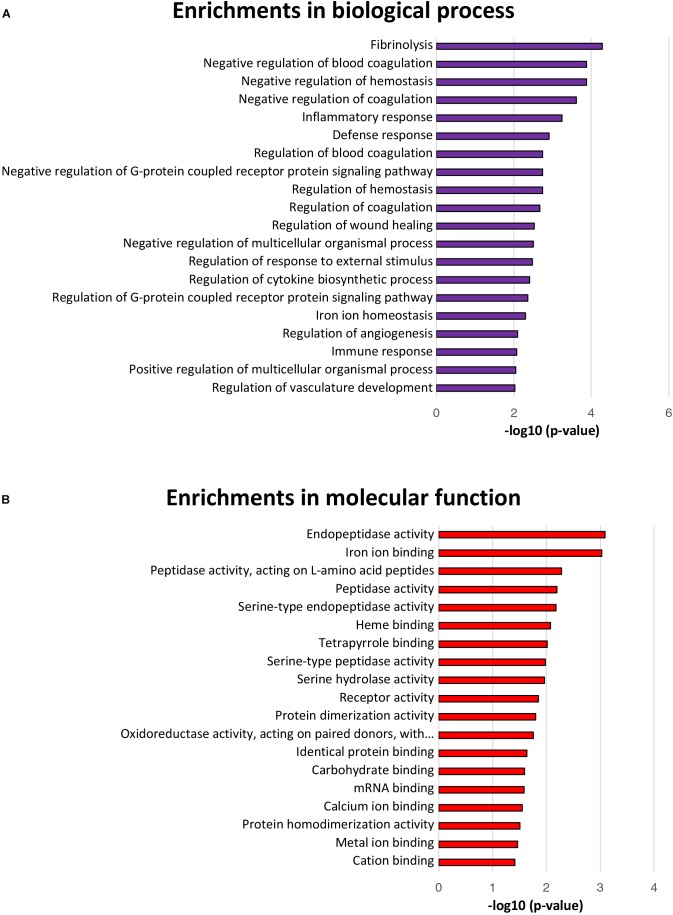
Biological processes and molecular function enrichment analyses for the differentially expressed proteins. **(A)** The top twenty significantly enriched GO terms for biological processes. The values in the column represent the normalized *p*-values (-log10). The most enriched biological process is displayed at the top of the column. **(B)** The significantly enriched GO terms for molecular function.

The GO terms for biological processes, including defense response, negative regulation of G-protein coupled receptor protein signaling pathway, regulation of response to external stimulus, regulation of G-protein coupled receptor protein signaling pathway, and iron ion homeostasis were significantly enriched, further suggesting a positive intestinal epithelial response to extracellular *L. gasseri* LA39 stimulus (Figure 4A). Several GO terms for molecular functions, including endopeptidase activity, peptidase activity, iron ion binding, heme binding, oxidoreductase activity acting on paired donors with the incorporation or reduction of molecular oxygen, metal ion binding, and cation binding were significantly enriched, suggesting a potential role of *L. gasseri* LA39 in the mitochondrial respiratory chain of IPEC-J2 cells (Figure 4B).

### Oxidative Phosphorylation (OXPHOS) Pathway in IPEC-J2 Cells Is Activated by the *L. gasseri* LA39

**FIGURE 5:**
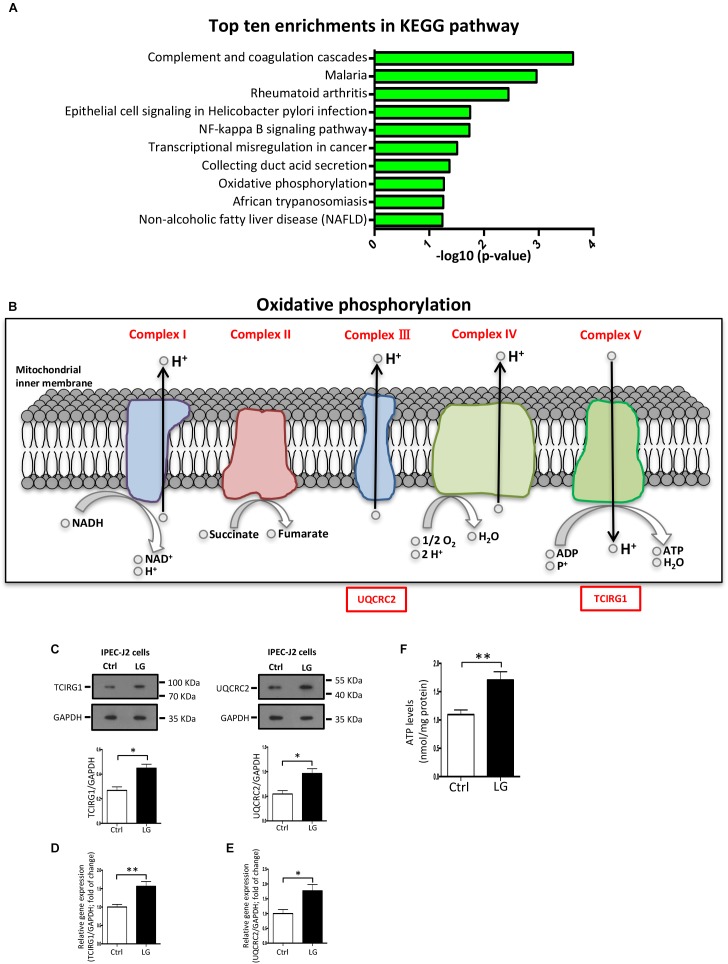
The OXPHOS pathway in porcine intestinal epithelial cells is activated by *L. gasseri* LA39. **(A)** KEGG pathway enrichment analysis for differentially expressed proteins. Values in the column indicate the normalized *p*-values (-log10). The most enriched KEGG pathway is displayed at the top of the column. **(B)** Differential expression profiles of proteins involved in OXPHOS metabolic pathway. All differentially expressed proteins are shown below the corresponding complex in OXPHOS pathway map. Protein marked with a red box showed a significantly increased expression comparing the *L. gasseri* LA39 group with the Ctrl group. **(C)** Western blotting analysis of the expression levels of TCIRG1, UQCRC2, and GAPDH in IPEC-J2 cells. Normalization and quantitation of TCIRG1/GAPDH and UQCRC2/GAPDH are shown in the corresponding bar chart. **(D,E)** The relative mRNA expression of TCIRG1, UQCRC2, and GAPDH in IPEC-J2 cells. Normalization and quantitation of TCIRG1/GAPDH **(D)** and UQCRC2/GAPDH **(E)** were shown in corresponding bar chart. **(F)** ATP levels of IPEC-J2 cells. The ATP concentration (nmol/L) was normalized to the total protein concentration (mg/L) of WCLs. Data are shown as mean ± SEM; *n* = 3 (C); *n* = 5 (E); *n* = 6 (F). ^∗∗^*p* < 0.01, ^∗^*p* < 0.05.

The KEGG pathway enrichment analysis of differentially expressed proteins was used to uncover the biological events in IPEC-J2 cells preferentially affected by *L. gasseri* LA39 treatment. The results indicated that several KEGG pathways, including complement and coagulation cascades, NF-kappa B signaling pathway, and OXPHOS were enriched (Figure 5A). Importantly, the expression levels of the differentially expressed proteins prothrombin, urokinase-type plasminogen activator, and vitronectin, involved in the complement and coagulation cascades pathway, significantly increased with *L. gasseri* LA39 treatment, suggesting the activation of the intestinal immune defense system. The expression levels of two differentially expressed proteins interleukin-8 and urokinase-type plasminogen activator, involved in the NF-kappa B signaling pathway, also significantly increased with *L. gasseri* LA39 treatment, indicating that the NF-kappa B signaling pathway in the intestinal epithelial cells was activated by *L. gasseri* LA39. Strikingly, the expression levels of two differentially expressed proteins UQCRC2 and TCIRG1, involved with the OXPHOS pathway, were significantly increased by *L. gasseri* LA39 treatment (Figure 5B). Combining the COG annotation and GO annotation analyses, the KEGG pathways analyses further suggested that the intestinal epithelial cellular mitochondrion functions, especially the OXPHOS pathway, might be preferentially affected by *L. gasseri* LA39 treatment. We further conducted the western blotting assay to validate the expression levels of the two differentially expressed proteins UQCRC2 and TCIRG1. The results demonstrated that the expression levels of UQCRC2 and TCIRG1 were significantly up-regulated upon *L. gasseri* LA39 treatment, which was consistent with the results of the iTRAQ analysis (Figure 5C). Our data also showed that the relative mRNA expression levels of UQCRC2 and TCIRG1 were both significantly up-regulated with *L. gasseri* LA39 treatment (Figures 5D,E). Given that ATP, the major cellular energy source, is mainly produced by the OXPHOS, we then measured the levels of cellular ATP. The results demonstrated that the levels of ATP in IPEC-J2 cells were significantly increased by *L. gasseri* LA39 treatment (Figure 5F). These findings indicated that *L. gasseri* LA39 activates the OXPHOS pathway, resulting in an increase in the energy production in IPEC-J2 cells.

### Oral Administration of *L. gasseri* LA39 Activates the OXPHOS Pathway in Intestinal Epithelial Cells of Early-Weaned Piglets

**FIGURE 6:**
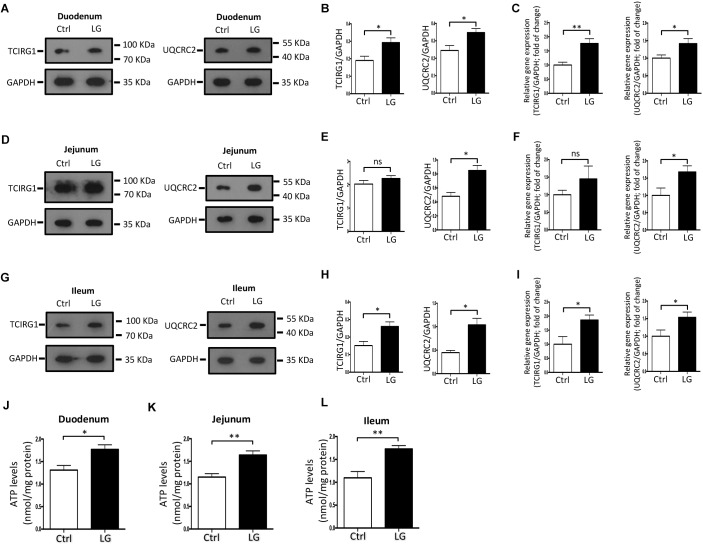
Oral administration of *L. gasseri* LA39 activates OXPHOS pathway and increases ATP levels in the intestinal epithelial cells of early-weaned piglets. **(A–C)** Western blotting analysis for the expression levels of TCIRG1, UQCRC2, and GAPDH in the duodenal epithelial cells **(A)**. Normalization and quantitation of TCIRG1/GAPDH and UQCRC2/GAPDH are shown in the corresponding bar chart **(B)**. **(C)** The relative mRNA expression levels of TCIRG1, UQCRC2, and GAPDH in duodenal epithelial cells. Normalization and quantitation of TCIRG1/GAPDH and UQCRC2/GAPDH were shown in corresponding bar chart. **(D–F)** Western blotting analysis for the expression levels of TCIRG1, UQCRC2, and GAPDH in the jejunal epithelial cells **(D)**. Normalization and quantitation of TCIRG1/GAPDH and UQCRC2/GAPDH are shown in the corresponding bar chart **(E)**. **(F)** The relative mRNA expression levels of TCIRG1, UQCRC2, and GAPDH in jejunal epithelial cells. Normalization and quantitation of TCIRG1/GAPDH and UQCRC2/GAPDH were shown in corresponding bar chart. **(G–I)** Western blotting analysis for the expression levels of TCIRG1, UQCRC2, and GAPDH in the ileal epithelial cells **(G)**. Normalization and quantitation of TCIRG1/GAPDH and UQCRC2/GAPDH are shown in the corresponding bar chart **(H)**. **(I)** The relative mRNA expression levels of TCIRG1, UQCRC2, and GAPDH in ileal epithelial cells. Normalization and quantitation of TCIRG1/GAPDH and UQCRC2/GAPDH were shown in corresponding bar chart. **(J–L)** ATP levels of intestinal epithelial cells, including duodenum **(J)**, jejunum **(K)**, and ileum **(L)**. The ATP concentration (nmol/L) was normalized to the total protein concentration (mg/L) of WCLs. Data are shown as mean ± SEM; *n* = 3 **(B,E,H)**; *n* = 5 **(C,F,I,J–L)**. ^∗∗^*p* < 0.01, ^∗^*p* < 0.05, and ns, not significant.

Based on the regulatory role of *L. gasseri* LA39 on the cultured IPEC-J2 cells, we next investigated the role of *L. gasseri* LA39 in the intestinal epithelial cells of weaned piglets *in vivo.* Our data indicated that oral gavage of *L. gasseri* LA39 significantly increased the expression levels and relative mRNA expression levels of UQCRC2 and TCIRG1 in the intestinal epithelial cells [including duodenum (Figures 6A–C), jejunum (Figures 6D–F), and ileum (Figures 6G–I), respectively]. The ATP levels in the intestinal epithelial cells were also significantly increased by oral gavage of *L. gasseri* LA39 (Figures 6J–L). These findings further indicate that *L. gasseri* LA39 can activate the OXPHOS pathway, resulting in an increase in the energy production in intestinal epithelial cells of weaned piglets. These findings suggest that *L. gasseri* LA39 may be used in intestinal epithelial energy production improvement in piglets upon early weaning stress.

## Discussion

In this study, we reported an important role of *L. gasseri* LA39 in OXPHOS pathway activation in the intestinal epithelial cells of piglets. Our findings suggest that *L. gasseri* LA39 may be used in increasing the intestinal energy production in mammals. Increasing evidence has linked the intestinal microbiota to intestinal epithelial protein expression regulation (Olivares et al., 2012; Siciliano and Mazzeo, 2012; van de Guchte et al., 2012; Luo et al., 2013; Ayllón et al., 2017). This study presents evidence that the intestinal epithelial OXPHOS pathway is significantly activated by *L. gasseri* LA39 and facilitates our understanding about the regulatory role of the intestinal microbes in the intestinal epithelium.

The present study used a canonical *in vitro* model of intestinal microbial interaction with host epithelium to evaluate the regulatory role of *L. gasseri* LA39 on the protein expression profiles in the porcine intestinal epithelial cell. Such an *in vitro* model of the intestinal epithelium-microbe interaction has been regarded as an efficient approach that has several advantages, including direct-contact co-cultures, simplicity, and convenience (Parlesak et al., 2004; van Nuenen et al., 2005). However, the intestinal epithelium-microbial co-culture approach may be limited in some experiments involving strict anaerobic microbes, which are unable to survive under aerobic conditions (Kim et al., 2012, 2016). The *L. gasseri* LA39 used in the present study is a facultative anaerobe and can also survive under aerobic conditions; thereby such an *in vitro* model can be appropriately used to investigate the intestinal epithelial responses to *L. gasseri* LA39. Our data revealed a significant alteration in the protein expression profiles of the intestinal epithelium by *L. gasseri* LA39 treatment, further suggesting the efficiency of this *in vitro* model in determining the intestinal epithelium-microbe interaction.

This study used the iTRAQ strategy to investigate the intestinal epithelial responses to *L. gasseri* LA39. Many studies have used proteomics to evaluate the intestinal epithelium-microbe interaction (Olivares et al., 2012; Siciliano and Mazzeo, 2012; van de Guchte et al., 2012; Luo et al., 2013; Ayllón et al., 2017). However, due to the limited identification capacity of traditional 2-DE-based proteomics strategies (Sun et al., 2012), investigating the integral effect of intestinal microbes on intestinal epithelium using a more advanced method will be of great significance. Recently, iTRAQ has been widely used to investigate the alteration of protein expression profiles due to its several advantages, including high sensitivity, high-throughput, and accuracy (Treumann and Thiede, 2010). Our previous study has revealed that leucine supplementation in diet induces an energy metabolism switch from OXPHOS toward glycolysis in intestinal epithelial cells of the weaned piglets, using iTRAQ strategy (Hu et al., 2017). However, the iTRAQ strategy has not been used to investigate the effects of microbes on the intestinal epithelium. Thus, the present study provides a new idea about the method for the intestinal epithelium-microbes interaction research in the future.

Our data revealed that *L. gasseri* LA39 can activate the OXPHOS pathway and increase the energy production in the intestinal epithelium of piglets both *in vitro* and *in vivo*. ATP, the major cellular energy source, is generated by both OXPHOS pathway and glycolysis pathway (Vander Heiden et al., 2009). Importantly, the OXPHOS pathway shows the maximum efficacy for producing the cellular ATP. According to the OXPHOS pathways, UQCRC2 protein belongs to the complex V in respiratory chain. It’s known to us that complex V has the function in producing the gradient of protons (or hydrogen ions) between two sides of the inner mitochondrial membrane, which is the essential basis for ATP production (Saada, 2014). According to the OXPHOS pathways, the TCIRG1 protein belongs to the complex V in respiratory chain. It’s known to us that complex V has the function in producing ATP (Saada, 2014). Thus, it’s reasonable that the *L. gasseri* LA39-mediated up-regulations in TCIRG1 and UQCRC2 proteins expression justify the increase in ATP levels. To our knowledge, there is no report about the regulation of the intestinal epithelial OXPHOS pathway by the intestinal microbes, though our previous study has revealed that dietary leucine supplementation induces an energy metabolism switch from OXPHOS toward glycolysis in the intestinal epithelial cells of the weaned piglets (Hu et al., 2017). Thus, for the first time, our data indicated that *L. gasseri* LA39 activates the OXPHOS pathway and increase the energy production in the intestinal epithelium of piglets, further suggesting an important role of the intestinal epithelium-microbe interaction on host health. Early weaning strategy, which facilitates the shortening of the cycle of slaughter in piglets and improves the reproductive performance in sows, has been widely used in pig production (Campbell et al., 2013). However, early weaning strategy induced a transformation in the diet from liquid milk toward solid feed, which causes a reduction in feed intake and intestinal indigestion in early-weaned piglets (Lalles et al., 2007). These adverse factors will cause an insufficient energy supply, thereby decreasing the growth and increasing the intestinal damages in early-weaned piglets. Thus, *L. gasseri* LA39 may be a probiotic candidate functioning in intestinal energy production promotion in early-weaned piglets. These results also suggest that *L. gasseri* LA39 may contribute to the intestinal energy production in human beings (especially infants) because of the similarities in the anatomy and nutritional physiology between pigs (*Sus scrofa*) and human beings (Vodicka et al., 2005; Maxmen, 2012; Meurens et al., 2012; Nielsen et al., 2014). The results also indicated that several KEGG pathways, including complement and coagulation cascades and NF-kappa B signaling pathway were enriched. Importantly, the expression levels of the differentially expressed proteins prothrombin, urokinase-type plasminogen activator, and vitronectin, involved in the complement and coagulation cascades pathway, significantly increased with *L. gasseri* LA39 treatment, suggesting the activation of the intestinal immune defense system. The expression levels of two differentially expressed proteins interleukin-8 and urokinase-type plasminogen activator, involved in the NF-kappa B signaling pathway, also significantly increased with *L. gasseri* LA39 treatment, indicating that the NF-kappa B signaling pathway in the intestinal epithelial cells was activated by *L. gasseri* LA39. These findings further suggest that the regulatory role of *L. gasseri* LA39 in intestinal epithelial cells is of complexity and many biological events need to be further explored, though we mainly focused on the OXPHOS pathway.

## Conclusion

In conclusion, our data revealed that *L. gasseri* LA39 activates the OXPHOS pathway and significantly increases the ATP levels in the porcine intestinal epithelial cells *in vitro* and *in vivo*. These findings suggest an important role of *L. gasseri* LA39 in increasing intestinal the energy production and the interactions of gut microbes with host physiology in mammals.

## Author Contributions

JH, LM, and XY designed the study. JH, LM, WZ, and YN conducted the experiments. JH, LM, and XY wrote the paper with the help of all the authors. All authors read and approved the final version of the manuscript.

## Conflict of Interest Statement

The authors declare that the research was conducted in the absence of any commercial or financial relationships that could be construed as a potential conflict of interest.

## References

[B1] Ayllón N. Jimenez-Marin A. Arguello H. Zaldivar-Lopez S. Villar M. Aguilar C. (2017). Comparative proteomics reveals differences in host-pathogen interaction between infectious and commensal relationship with *Campylobacter jejuni*. *Front. Cell. Infect. Microbiol.* 7:145. 10.3389/fcimb.2017.00145 28491823 PMC5405767

[B2] Backhed F. Manchester J. K. Semenkovich C. F. Gordon J. I. (2007). Mechanisms underlying the resistance to diet-induced obesity in germ-free mice. *Proc. Natl. Acad. Sci. U.S.A.* 104 979–984. 10.1073/pnas.0605374104 17210919 PMC1764762

[B3] Borody T. J. Khoruts A. (2011). Fecal microbiota transplantation and emerging applications. *Nat. Rev. Gastroenterol. Hepatol.* 9 88–96. 10.1038/nrgastro.2011.244 22183182

[B4] Campbell J. M. Crenshaw J. D. Polo J. (2013). The biological stress of early weaned piglets. *J. Anim. Sci. Biotechnol.* 4:19. 10.1186/2049-1891-4-19 23631414 PMC3651348

[B5] Fan Q. Long B. Yan G. Wang Z. Shi M. Bao X. (2017). Dietary leucine supplementation alters energy metabolism and induces slow-to-fast transitions in *longissimus dorsi* muscle of weanling piglets. *Br. J. Nutr.* 117 1222–1234. 10.1017/s0007114517001209 28643619

[B6] Hill C. Guarner F. Reid G. Gibson G. R. Merenstein D. J. Pot B. (2014). Expert consensus document. The International Scientific Association for Probiotics and Prebiotics consensus statement on the scope and appropriate use of the term probiotic. *Nat. Rev. Gastroenterol. Hepatol.* 11 506–514. 10.1038/nrgastro.2014.66 24912386

[B7] Hu J. Chen L. Zheng W. Shi M. Liu L. Xie C. (2018). *Lactobacillus frumenti* facilitates intestinal epithelial barrier function maintenance in early-weaned piglets. *Front. Microbiol.* 9:897. 10.3389/fmicb.2018.00897 29867808 PMC5958209

[B8] Hu Z. Du M. Lai W. Liang Y. Liu Q. Mo Y. (2018). Energy metabolism in the bone is associated with histomorphometric changes in rats with hyperthyroidism. *Cell. Physiol. Biochem.* 46 1471–1482. 10.1159/000489187 29689555

[B9] Hu J. Nie Y. Chen J. Zhang Y. Wang Z. Fan Q. (2016). Gradual changes of gut microbiota in weaned miniature piglets. *Front. Microbiol.* 7:1727. 10.3389/fmicb.2016.01727 27853453 PMC5090779

[B10] Hu J. Nie Y. Chen S. Xie C. Fan Q. Wang Z. (2017). Leucine reduces reactive oxygen species levels via an energy metabolism switch by activation of the mTOR-HIF-1alpha pathway in porcine intestinal epithelial cells. *Int. J. Biochem. Cell. Biol.* 89 42–56. 10.1016/j.biocel.2017.05.026 28583467

[B11] Huang H. Y. Zhang W. T. Jiang W. Y. Chen S. Z. Liu Y. Ge X. (2015). RhoGDIbeta inhibits bone morphogenetic protein 4 (BMP4)-induced adipocyte lineage commitment and favors smooth muscle-like cell differentiation. *J. Biol. Chem.* 290 11119–11129. 10.1074/jbc.M114.608075 25778399 PMC4409270

[B12] Huang Y. Liao M. Yang Q. Xiao J. Hu Z. Cao H. (2018). iTRAQ-based quantitative proteome revealed metabolic changes of *Sitophilus zeamais* in response to terpinen-4-ol fumigation. *Pest Manag. Sci.* 10.1002/ps.5135 [Epub ahead of print]. 30039555

[B13] Ivanov I. I. Atarashi K. Manel N. Brodie E. L. Shima T. Karaoz U. (2009). Induction of intestinal Th17 cells by segmented filamentous bacteria. *Cell* 139 485–498. 10.1016/j.cell.2009.09.033 19836068 PMC2796826

[B14] Kawai Y. Ishii Y. Uemura K. Kitazawa H. Saito T. Itoh T. (2001). *Lactobacillus reuteri* LA6 and *Lactobacillus gasseri* LA39 isolated from faeces of the same human infant produce identical cyclic bacteriocin. *Food Microbiol.* 18 407–415. 10.1006/fmic.2001.0412

[B15] Kawai Y. Saito T. Toba T. Samant S. K. Itoh T. (1994). Isolation and characterization of a highly hydrophobic new bacteriocin (gassericin A) from *Lactobacillus gasseri* LA39. *Biosci. Biotechnol. Biochem.* 58 1218–1221. 10.1271/bbb.58.1218 7765246

[B16] Kim H. J. Huh D. Hamilton G. Ingber D. E. (2012). Human gut-on-a-chip inhabited by microbial flora that experiences intestinal peristalsis-like motions and flow. *Lab Chip* 12 2165–2174. 10.1039/c2lc40074j 22434367

[B17] Kim H. J. Li H. Collins J. J. Ingber D. E. (2016). Contributions of microbiome and mechanical deformation to intestinal bacterial overgrowth and inflammation in a human gut-on-a-chip. *Proc. Natl. Acad. Sci. U.S.A.* 113 E7–E15. 10.1073/pnas.1522193112 26668389 PMC4711860

[B18] Kim M. Ashida H. Ogawa M. Yoshikawa Y. Mimuro H. Sasakawa C. (2010). Bacterial interactions with the host epithelium. *Cell Host Microbe* 8 20–35. 10.1016/j.chom.2010.06.006 20638639

[B19] Kleerebezem M. Hols P. Bernard E. Rolain T. Zhou M. Siezen R. J. (2010). The extracellular biology of the lactobacilli. *FEMS. Microbiol. Rev.* 34 199–230. 10.1111/j.1574-6976.2010.00208.x 20088967

[B20] Lalles J. P. Bosi P. Smidt H. Stokes C. R. (2007). Nutritional management of gut health in pigs around weaning. *Proc. Nutr. Soc.* 66 260–268. 10.1017/s0029665107005484 17466106

[B21] Lemon K. P. Armitage G. C. Relman D. A. Fischbach M. A. (2012). Microbiota-targeted therapies: an ecological perspective. *Sci. Transl. Med.* 4:137rv135. 10.1126/scitranslmed.3004183 22674555 PMC5725196

[B22] Li M. Hui X. Pan Y. Cai H. Dai Y. Xu X. (2018). Proteomic evaluation of human umbilical cord tissue exposed to polybrominated diphenyl ethers in an e-waste recycling area. *Environ. Int.* 111 362–371. 10.1016/j.envint.2017.09.016 29169793

[B23] Long B. Yin C. Fan Q. Yan G. Wang Z. Li X. (2016). Global liver proteome analysis using iTRAQ reveals AMPK-mTOR-autophagy signaling is altered by intrauterine growth restriction in newborn piglets. *J. Proteome Res.* 15 1262–1273. 10.1021/acs.jproteome.6b00001 26967195

[B24] Luo J. Zheng A. Meng K. Chang W. Bai Y. Li K. (2013). Proteome changes in the intestinal mucosa of broiler (*Gallus gallus*) activated by probiotic *Enterococcus faecium*. *J. Proteomics* 91 226–241. 10.1016/j.jprot.2013.07.017 23899589

[B25] Maxmen A. (2012). Model pigs face messy path. *Nature* 486:453. 10.1038/486453a 22739291

[B26] Meurens F. Summerfield A. Nauwynck H. Saif L. Gerdts V. (2012). The pig: a model for human infectious diseases. *Trends Microbiol.* 20 50–57. 10.1016/j.tim.2011.11.002 22153753 PMC7173122

[B27] Nielsen K. L. Hartvigsen M. L. Hedemann M. S. Laerke H. N. Hermansen K. Bach Knudsen K. E. (2014). Similar metabolic responses in pigs and humans to breads with different contents and compositions of dietary fibers: a metabolomics study. *Am. J. Clin. Nutr.* 99 941–949. 10.3945/ajcn.113.074724 24477039

[B28] Olivares M. Laparra M. Sanz Y. (2012). Oral administration of *Bifidobacterium longum* CECT 7347 modulates jejunal proteome in an in vivo gliadin-induced enteropathy animal model. *J. Proteomics* 77 310–320. 10.1016/j.jprot.2012.09.005 23023000

[B29] Parlesak A. Haller D. Brinz S. Baeuerlein A. Bode C. (2004). Modulation of cytokine release by differentiated CACO-2 cells in a compartmentalized coculture model with mononuclear leucocytes and nonpathogenic bacteria. *Scand. J. Immunol.* 60 477–485. 10.1111/j.0300-9475.2004.01495.x 15541040

[B30] Ren Y. Hao P. Dutta B. Cheow E. S. Sim K. H. Gan C. S. (2013). Hypoxia modulates A431 cellular pathways association to tumor radioresistance and enhanced migration revealed by comprehensive proteomic and functional studies. *Mol. Cell. Proteomics* 12 485–498. 10.1074/mcp.M112.018325 23204318 PMC3567868

[B31] Rhein V. Song X. Wiesner A. Ittner L. M. Baysang G. Meier F. (2009). Amyloid-beta and tau synergistically impair the oxidative phosphorylation system in triple transgenic Alzheimer’s disease mice. *Proc. Natl. Acad. Sci. U.S.A.* 106 20057–20062. 10.1073/pnas.0905529106 19897719 PMC2774257

[B32] Saada A. (2014). Mitochondria: mitochondrial OXPHOS (dys) function ex vivo–the use of primary fibroblasts. *Int. J. Biochem. Cell. Biol.* 48 60–65. 10.1016/j.biocel.2013.12.010 24412346

[B33] Savitski M. Wilhelm M. Hahne H. Kuster B. Bantscheff M. (2015). A scalable approach for protein false discovery rate estimation in large proteomic data sets. *Mol. Cell. Proteomics* 14 2394–2404. 10.1074/mcp.M114.046995 25987413 PMC4563723

[B34] Shi Q. Chen L. N. Zhang B. Y. Xiao K. Zhou W. Chen C. (2015). Proteomics analyses for the global proteins in the brain tissues of different human prion diseases. *Mol. Cell. Proteomics* 14 854–869. 10.1074/mcp.M114.038018 25616867 PMC4390265

[B35] Siciliano R. A. Mazzeo M. F. (2012). Molecular mechanisms of probiotic action: a proteomic perspective. *Curr. Opin. Microbiol.* 15 390–396. 10.1016/j.mib.2012.03.006 22538051

[B36] Sommer F. Backhed F. (2013). The gut microbiota–masters of host development and physiology. *Nat. Rev. Microbiol.* 11 227–238. 10.1038/nrmicro2974 23435359

[B37] Sun H. Li M. Gong L. Liu M. Ding F. Gu X. (2012). iTRAQ-coupled 2D LC-MS/MS analysis on differentially expressed proteins in denervated tibialis anterior muscle of *Rattus norvegicus*. *Mol. Cell. Biochem.* 364 193–207. 10.1007/s11010-011-1218-2 22227918

[B38] Treumann A. Thiede B. (2010). Isobaric protein and peptide quantification: perspectives and issues. *Expert Rev. Proteomics* 7 647–653. 10.1586/epr.10.29 20973638

[B39] van Baarlen P. Wells J. M. Kleerebezem M. (2013). Regulation of intestinal homeostasis and immunity with probiotic lactobacilli. *Trends Immunol.* 34 208–215. 10.1016/j.it.2013.01.005 23485516

[B40] van de Guchte M. Chaze T. Jan G. Mistou M. Y. (2012). Properties of probiotic bacteria explored by proteomic approaches. *Curr. Opin. Microbiol.* 15 381–389. 10.1016/j.mib.2012.04.003 22658701

[B41] van Nuenen M. H. de Ligt R. A. Doornbos R. P. van der Woude J. C. Kuipers E. J. Venema K. (2005). The influence of microbial metabolites on human intestinal epithelial cells and macrophages in vitro. *FEMS. Immunol. Med. Microbiol.* 45 183–189. 10.1016/j.femsim.2005.03.010 15939578

[B42] Vander Heiden M. Cantley L. Thompson C. (2009). Understanding the Warburg effect: the metabolic requirements of cell proliferation. *Science* 324 1029–1033. 10.1126/science.1160809 19460998 PMC2849637

[B43] Ventura M. O’Flaherty S. Claesson M. J. Turroni F. Klaenhammer T. R. van Sinderen D. (2009). Genome-scale analyses of health-promoting bacteria: probiogenomics. *Nat. Rev. Microbiol.* 7 61–71. 10.1038/nrmicro2047 19029955

[B44] Vodicka P. Smetana K. Jr. Dvoránková B. Emerick T. Xu Y. Z. Ourednik J. (2005). The miniature pig as an animal model in biomedical research. *Ann. N. Y. Acad. Sci.* 1049 161–171. 10.1196/annals.1334.015 15965115

[B45] Wang H. Wei H. Tang L. Lu J. Mu C. Wang C. (2018). A proteomics of gills approach to understanding salinity adaptation of *Scylla paramamosain*. *Gene* 677 119–131. 10.1016/j.gene.2018.07.059 30055305

[B46] Weatherly D. Atwood J. Minning T. Cavola C. Tarleton R. Orlando R. (2005). A Heuristic method for assigning a false-discovery rate for protein identifications from Mascot database search results. *Mol. Cell. Proteomics* 4 762–772. 10.1074/mcp.M400215-MCP200 15703444

[B47] Wen B. Zhou R. Feng Q. Wang Q. Wang J. Liu S. (2014). IQuant: an automated pipeline for quantitative proteomics based upon isobaric tags. *Proteomics* 14 2280–2285. 10.1002/pmic.201300361 25069810

[B48] Zhu M. Dai S. McClung S. Yan X. Chen S. (2009). Functional differentiation of *Brassica napus* guard cells and mesophyll cells revealed by comparative proteomics. *Mol. Cell. Proteomics* 8 752–766. 10.1074/mcp.M800343-MCP200 19106087 PMC2667361

